# HIV and Pregnancy: Navigating Complex Decision Making and Preventing Perinatal Transmission

**DOI:** 10.1007/s11904-025-00745-0

**Published:** 2025-06-07

**Authors:** William R. Short, Elizabeth D. Lowenthal, Florence Momplaisir, Kathleen M. Powis, Rachel K. Scott, Lynn M. Yee, Emily S. Miller, Lynne M. Mofenson

**Affiliations:** 1 Department of Medicine, Division of Infectious Diseases, Perelman School of Medicine, University of Pennsylvania, 3801 Filbert St, Suite 103B, Philadelphia, PA 19104 USA; 2 Department of Pediatrics, Perelman School of Medicine, University of Pennsylvania, Philadelphia, PA USA; 3 Department of Biostatistics, Epidemiology, and Informatics, Perelman School of Medicine, University of Pennsylvania, Philadelphia, PA USA; 4 Children’s Hospital of Philadelphia, Philadelphia, PA USA; 5 Leonard Davis Institute of Health Economics, University of Pennsylvania, Philadelphia, PA USA; 6 Department of Internal Medicine and Pediatrics, Massachusetts General Hospital, Boston, MA USA; 7 Department of Immunology and Infectious Diseases, Harvard T.H. Chan School of Public Health, Boston, MA USA; 8 Medstar Health and Georgetown University School of Medicine, Washington, DC USA; 9 Department of Obstetrics and Gynecology, Division of Maternal Fetal Medicine, Northwestern University Feinberg School of Medicine, Chicago, IL USA; 10 Department of Obstetrics and Gynecology, Division of Maternal Fetal Medicine, Warren Alpert Medical School of Brown University, Providence, Rhode Island USA; 11 Elizabeth Glaser Pediatric AIDS Foundation, Washington, DC USA

**Keywords:** HIV, Antiretroviral therapy, Pregnancy, Teratogenicity

## Abstract

**Purpose of Review:**

The objective of this review is to examine the intersection of pregnancy and HIV, focusing on birthing person and fetal health outcomes, prevention of perinatal HIV transmission, and the latest advancements in treatment and care in the United States. It highlights current guidelines, challenges in management, and future directions for improving outcomes.

**Recent Findings:**

HIV treatment guidelines continue to highlight key principles for the choice of antiretroviral therapy in pregnancy, challenges, and strategies for adherence support. Guidelines have been updated to reflect patient-centered counseling to support shared decision making about infant feeding. Counseling should begin prior to pregnancy, and be reviewed throughout pregnancy, again at delivery, and throughout the periods when breast/chestfeeding occurs.

**Summary:**

ART use during pregnancy has significantly reduced perinatal HIV transmission. Ongoing research and collaboration are vital to addressing remaining challenges. Prioritizing maternal and infant health ensures that ART not only prevents transmission but also improves future health for families affected by HIV.

## Introduction

**Fig. 1:**
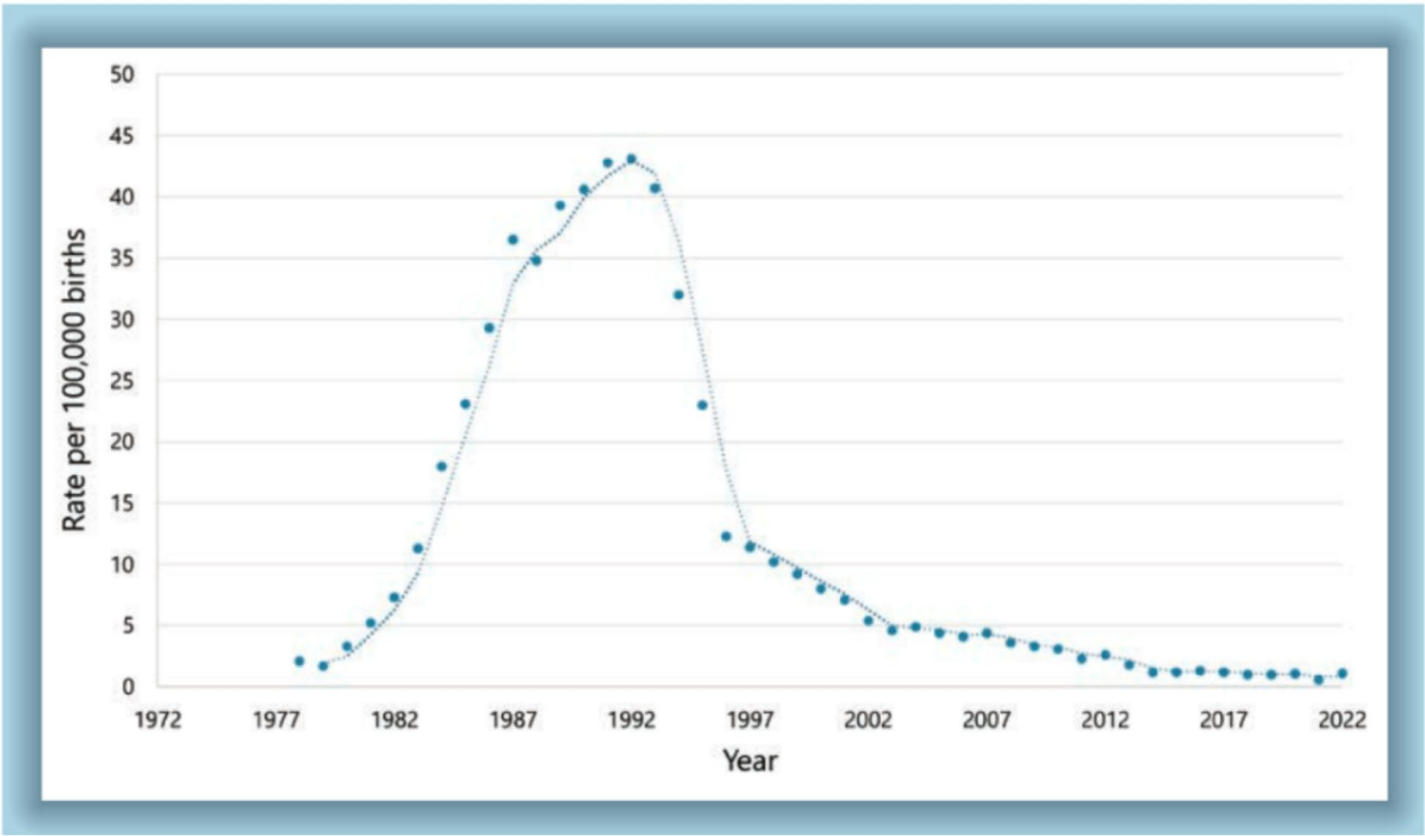
Rate of Perinatal HIV transmission over time. Reproduced with permission from Keeshin S and Ciaranello A. Eckel RH. HIV and pregnancy: treatment, infant feeding, and prevention. NEJM group infectious diseases update October 2024, pp. 15–19, Copyright 2024 Massachusetts Medical Society

Over the past three decades, perinatal transmission of HIV-1 has dramatically decreased in resource rich settings such as the United States (US), largely due both to the widespread availability of antiretroviral therapy (ART) and to seismic advances in the effectiveness and tolerability of ART regimens [1]. These interventions have dramatically reduced the risk of transmission to less than 2% in high-income countries, marking a major public health achievement (see Fig. 1).

Although prevention of perinatal transmission is still prioritized in prenatal care, there has been a paradigm shift to not only stabilize and optimize maternal health and minimize risk of transmission, but to offer holistic care to birthing persons living with HIV (PLWH), a chronic condition. PLWH are more likely to have a lifetime history of trauma and behavioral health comorbidities, such as post-traumatic stress disorder [2], depression [3], anxiety [3], and substance use disorder (SUD) [4, 5], and these comorbidities may persist in or be exacerbated during pregnancy. Addressing comorbid conditions is integral both to comprehensive care as well as ART adherence and retention in care. In this paper, we will review advances in ART, neonatal HIV prophylaxis/treatment, SUD in pregnancy, maternal behavioral health, postpartum retention in care, and infant feeding in the setting of maternal HIV.

## Choice of Antiretroviral Therapy

ART has revolutionized the management of HIV, transforming a once-terminal illness into a chronic manageable condition. As a result of these advances, PLWH may decide to start or expand their families. Management of persons with chronic conditions often requires medication to maintain their health, which can present unique challenges during pregnancy [6]. Many drugs are insufficiently studied for safety and effectiveness data in pregnancy, forcing clinicians to choose between older, less effective options or continuing newer medications with limited data. This dilemma is especially critical for antiretroviral (ARV) medications used to manage HIV.

For an ARV drug to be recommended for use during pregnancy by national guidelines, three key data points must be evaluated: efficacy, pharmacokinetics (PK), and safety [7]. This level of evidence ensures the HIV treatment is effective and minimizes the fetal exposure to potentially harmful drugs. Unfortunately, pregnant persons are often excluded from pre-approval studies of novel therapeutics unless the drug is intended specifically for pregnancy, creating a significant gap in data for pregnant persons with HIV [8].

Because the course of HIV disease and effects of ARVs is similar in pregnant and non-pregnant populations, ARV efficacy data can be extrapolated from studies conducted on nonpregnant populations. However, PK data specific to pregnancy remain scarce. Pregnancy-induced physiological changes affect drug metabolism, yet PK studies for ARVs are often delayed by years due to limited funding and greater complexity of these studies, including the need to evaluate both maternal and fetal exposures and the recognition that pregnancy is a dynamic process that changes as gestational age advances. On average, a median of six years passes between Food and Drug Administration (FDA) approval of an ARV and the first PK data in pregnancy are published [8].

Data on drug safety during pregnancy come from various sources, including animal reproductive-toxicity studies, case reports, and registries. However, pre-clinical animal data often fail to predict human outcomes [9]. To address this gap, timely collection of unbiased, prospective data on new ARVs is essential. The Antiretroviral Pregnancy Registry (APR), established in 1989, is a vital resource for collecting data on ARV exposure during pregnancy. It relies on voluntary, prospective reporting from healthcare providers, meaning cases must be registered before the pregnancy outcome is known to reduce bias [10]. The APR uses predefined methods to analyze data, including prospectively collecting birth outcomes reported by providers and reporting on them when at least 200 infants have been exposed to an ARV in the first trimester. A cohort of 200 exposures is required to detect a doubling of the risk compared to the population-based expected rate for the overall prevalence of birth defects (3%) with 80% power and a Type I error rate of 5% [11]. However, like PK data, it takes a median of six years from FDA approval to the first published safety data on ARV use during pregnancy in the APR [12].

Monitoring for an association of drug exposure with rare events such as neural tube defects (NTDs) require data on large numbers of individuals with exposure to the drug. Historical examples demonstrate the importance of timely reporting and collection of pregnancy exposure data. Concern was raised about potential fetal central nervous system abnormalities including NTDs from initial pre-clinical primate studies and a small number of early human case reports of efavirenz (EFV) exposure at conception, leading to recommendations against its use during pregnancy. However, after more than a decade of data collection, EFV exposure at conception was found not to increase the risk of NTDs [13–15]. Similarly, concerns about dolutegravir (DTG) and NTDs were raised by the Botswana Tsepamo birth surveillance study in an initial evaluation including less than 500 pre-conception DTG exposures, but with collection of data on much larger numbers of pre-conception DTG exposures in the Tsepamo study as well other birth surveillance studies, together providing information on over 14,000 DTG pre-conception exposures, it became evident that the initial finding was erroneous conclusion based on small numbers, with no association of pre-conception DTG and NTDs [16–19]. These findings influenced global policy, changed drug label information, and highlighted the critical role of ARV exposure reporting in shaping recommendations.

As new HIV treatments, such as long-acting injectable cabotegravir, rilpivirine, and lenacapravir become available, there is a critical need for pregnancy-specific pharmacokinetic data. Minimal data currently exist on cabotegravir or lenacapravir exposure during pregnancy, making it imperative for HIV care providers to contribute to registries like the APR, to prospectively capture outcomes as they occur. This collective effort is essential to ensure timely, evidence-based guidance for pregnant people with HIV.

Current guidelines recommend starting a backbone of two nucleoside reverse transcriptase inhibitors plus a third anchor drug of either dolutegravir or bictegravir for pregnant persons who have never received ART [7]. If there has been exposure to long-acting injectable cabotegravir for pre-exposure prophylaxis, the use of a boosted protease inhibitor regimen is recommended initially pending the results of genotype testing including testing for integrase inhibitor resistance. Table 1 highlights key recommendations by the US Panel on Treatment of HIV in Pregnancy and Prevention of Perinatal Transmission Guideline Panel.

**Table 1:** Antiretroviral therapy recommendations during pregnancy

**Pregnant people who have never received antiretroviral drugs**
• tenofovir disoproxil fumarate/tenofovir alafenamide + emtricitabine/lamivudine with dolutegravir • tenofovir alafenamide/emtricitabine/bictegravir
**Pregnant people who were receiving cabotegravir for Pre-exposure Prophylaxis**
• tenofovir disoproxil fumarate/tenofovir alafenamide + emtricitabine/lamivudine with darunavir/ritonavir* twice daily
Pregnant People who present on a non-preferred regimen **
People should be given information about making changes to an existing regimen and the decision individualized. They should continue with HIV viral load monitoring (every 1–2 months) or consider switching because of less data on non-preferred than preferred regimens
**Drug combinations not recommended**
• emtricitabine/tenofovir disoproxil fumarate/elvitegravir/cobicistat • emtricitabine/tenofovir alafenamide/elvitegravir/cobicistat • atazanavir/cobicistat • emtricitabine/tenofovir alafenamide/darunavir/cobicistat • darunavir/cobicistat

*Check HIV genotype (including testing for integrase inhibitor resistance) to determine need for continuation of boosted protease inhibitor

**Drugs such as LA-CAB/RPV or 2 drug regimens

## Infant Prophylaxis/Treatment

**Fig. 2:**
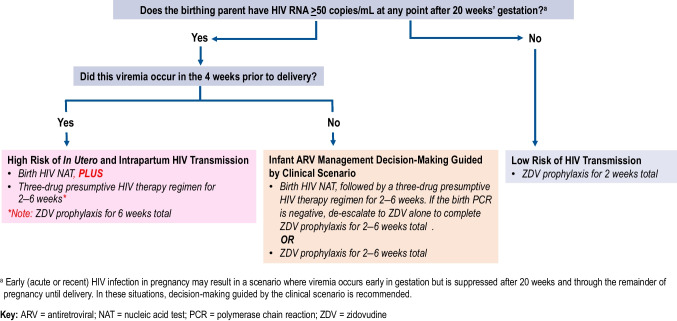
Algorithm for antiretroviral management of infants with In Utero or Intrapartum HIV exposure by risk of HIV transmission. Source: Panel on antiretroviral therapy and medical management of children living with HIV. Guidelines for the use of antiretroviral agents in pediatric Hiv Infection. Department of Health and Human Services. Available at https://clinicalinfo.hiv.gov/en/guidelines/pediatric-arv. Accessed April 10, 2025

All infants with perinatal (i.e., in utero, intrapartum, and/or lactation-associated) exposure to HIV are at risk of HIV acquisition. However, the degree of risk depends on the amount of HIV RNA to which the fetuses are exposed, as well as the timing of exposure. Appropriate prophylaxis or presumptive HIV therapy of a newborn whose birthing parent is living with HIV is determined based on assessment of the infant’s risk of HIV acquisition, with initiation occurring ideally within the first six hours after birth. An algorithm for assessing infant risk, created by the Panel on Antiretroviral Therapy and Medical Management of Children Living with HIV, is presented in Fig. 2. Current US recommendations based on maternal risk factors and the infant’s gestational age at birth are outlined in Table 2. When pregnant persons achieve sustained viral suppression, defined as at least two consecutive HIV RNA test results of < 50 copies/mL from 20 weeks gestation through delivery, infants born full-term (gestational age ≥ 37 weeks) are categorized as low risk under current US guidelines [7], with guidelines recommending 2 weeks of infant zidovudine prophylaxis.

**Table 2:** Recommended newborn antiretroviral prophylaxis and presumptive therapy

Risk Category	Medications	Duration
Low-risk term infants: • Newborn is ≥ 37 weeks gestation • Mother received antiretroviral therapy (ART) for at least last 10 weeks of pregnancy • Maternal HIV RNA < 50 copies/mL (2 consecutive tests ≥ 4 weeks apart) • No maternal primary or acute HIV infection during pregnancy No identified maternal ART adherence concerns	Zidovudine monoprophylaxis	2 weeks
High-risk term newborns: • No antepartum ART • Maternal HIV RNA not < 50 copies/mL within 4 weeks of delivery and at least 4 weeks before last RNA level • Maternal primary or acute HIV infection during pregnancy (including first positive HIV test during labor or delivery*) Or, term infants born to women who acquire primary or acute HIV during breastfeeding	Presumptive HIV therapy: • Nevirapine + zidovudine + (lamivudine or emtricitabine) • Raltegravir + zidovudine + (lamivudine or emtricitabine)	4–6 weeks + at least two negative HIV RNA tests) *(if on a nevirapine-based regimen, consider stopping NVP 1–2 weeks before other medicines)*

For infants at high risk for HIV acquisition under US guidelines (defined born to a pregnant person with HIV RNA ≥ 50 copies/mL in the 4 weeks prior to delivery, incident HIV infection during pregnancy, or not receiving ART), presumptive HIV therapy with three effective ARV medications is recommended since early treatment limits viral reservoirs and improves long-term outcomes should the infant prove to be infected. However, for ill and extremely premature infants who are unable to tolerate oral medications, zidovudine is still the only ARV which is available for intravenous delivery to infants. Infants with in utero or perinatal HIV exposure who do not meet low- or high- risk criteria require consideration of case-specific factors, with selection of the number of ARVs used and duration of use tailored to the level and timing of HIV viremia during the pregnancy.

## Perinatal Mental Health Among Pregnant Persons Living with HIV

Pregnancy and the postpartum period are high-risk periods for the emergence or exacerbation of mental health conditions [20, 21] Pregnant and postpartum PLWH face increased risks for mental health challenges due to the intersecting effects of biological, social, and structural factors [22, 23]. Stigma plays a central role in exacerbating mental health issues among pregnant and postpartum PLWH [24, 25]. Internalized HIV-related stigma has been linked to depressive symptoms and social isolation, further perpetuating poor mental health [26]. Additionally, intimate partner violence (IPV) remains a pervasive issue, affecting approximately 9% of pregnant PLWH in the US [27].

The prevalence of antenatal and postnatal depression in PLWH is estimated at 36–44% and 21–31%, respectively [28, 29]. Anxiety symptoms and disorders are also prevalent, driven by concerns over health outcomes and societal stigma [30]. Depression and anxiety during the perinatal period can significantly affect health behaviors, including prenatal care initiation and ART adherence [31]. Physiological and psychological stressors associated with HIV and pregnancy can also lead to poorer neonatal outcomes, as stress and mental health symptoms during pregnancy have been linked to adverse birth and child developmental outcomes. These challenges underscore the importance of addressing mental health as a component of comprehensive HIV care.

Despite the high prevalence of mental health conditions, pregnant and postpartum PLWH often face barriers to accessing mental health care [32]. Structural interventions and culturally sensitive mental health programs are urgently needed to address these gaps. Comprehensive care models, such as the collaborative care model, that integrate mental health services with routine HIV care are essential for addressing the complex needs of this population.

## Substance Use and Substance Use Disorder Among Pregnant PLWH

The HIV and substance use epidemics intersect in the US, and substance use by PLWH remains an important area for clinical and public health attention. Ending the HIV epidemic requires addressing substance use, which poses challenges to the HIV care continuum and has health implications for pregnant and non-pregnant PLWH. Among pregnant PLWH, rates of substance use were historically high, with nearly half reporting use of illicit substances in the 1980 s and 1990 s [33]. However, the substance use epidemic has changed alongside evolving epidemiology of HIV acquisition among cis-gendered women. Rough et al. demonstrated that from 1990–2012, there was a substantial decline in substance use during pregnancy among PLWH, with rates mirroring the general population by the end of the study period [34].

However, the opioid crisis in the US has made opioid use disorder, driven largely by use of prescription opioids, a growing problem among pregnant people and reproductive age females [35–39]. Opioid use and opioid use disorder have substantial implications for perinatal outcomes and long-term health [38]. Concurrent with the opioid epidemic, rates of cannabis use have risen in pregnant people in the context of increasing legalization [40–44], with 34–60% of people reporting continued use during pregnancy despite recommendations against its use during pregnancy or lactation [45]. Cannabis, in particular, has potential fetal growth and neurodevelopmental implications, and its use is often concomitant with other potentially harmful substances [34, 45–50]. These patterns of substance use and SUD also exist among pregnant PLWH. Data from the Pediatric HIV/AIDS Cohort Study (PHACS) demonstrated that from 2007 to 2019, opioid use among pregnant PLWH was low, but cannabis use in pregnancy and postpartum was common and rising over time, particularly in states with legalized medical cannabis use [51]. Other work from PHACS has demonstrated that there are no significant differences in opioid, alcohol, or cannabis use by pregnant or postpartum PWH by their mode of HIV acquisition, highlighting the importance of universal substance use screening and counseling [52].

Substance use and SUD are intricately linked to adverse social determinants of health and may compound other health and health care challenges experienced by pregnant PLWH [53]. Given the changing epidemiology of substance use among pregnant people and the unique circumstances experienced by PLWH, clinical care must include person-centered, nonjudgmental universal screening and counseling about the maternal and fetal harms of substance use and available treatment [39]. When treatment for SUD is indicated, a number of best practices for a pregnant population are applicable to PLWH [39]. In addition, substance use screening and care in all pregnant people, and especially PLWH, require consideration of reproductive and racial justice, given the many inequities and long history of punitive measures for historically minoritized pregnant people with substance use [54]. Finally, postpartum substance use also warrants clinical attention as a part of a comprehensive effort to improve interpregnancy and long-term health [55], particularly given the importance of ART adherence for maternal and neonatal well-being.

## Special Considerations for Adults with Perinatally Acquired HIV

Due to effective ART, many people with perinatally acquired HIV (PHIV) are reaching adulthood and achieving pregnancy. Significant challenges exist in managing pregnant people with PHIV due to the unique clinical considerations resulting from lifelong exposure to HIV, associated ART toxicity and co-morbidities, and ART treatment interruptions from treatment fatigue with concomitant drug resistance [56]. Although many people with PHIV are motivated to adhere to their ART to prevent transmission of HIV to their children, compared to people with non-PHIV, people with PHIV are more likely to experience HIV viremia in pregnancy due to a combination of clinical and psychosocial factors [57]. Prior drug resistance can limit the availability of simplified and well-tolerated ART regimens in pregnancy. In addition, studies show that people with PHIV may face increased risks of preterm birth, small-for-gestational-age infants, and cesarean deliveries due to elevated viral loads [58]. Postpartum, retention in care and viral suppression often decline, highlighting the importance of adherence interventions after childbirth [59].

Important psychosocial factors include lifelong HIV stigma [60] and cognitive impairment due to impacts of HIV and chronic inflammation on the brain [61] which can add complexities in prenatal care management. Additional challenges to HIV care prenatally for people with PHIV, such as depression, parental loss, and social or developmental challenges are common [62]. Depression is prevalent among pregnant people with PHIV and is linked to lower ART adherence rates [56]. Effective prenatal care for people with PHIV includes addressing mental health and providing developmentally appropriate adherence support. Pregnancy in adolescents or young adults with PHIV can complicate the transition from pediatric to adult HIV care [63], underscoring the need for structured support and care coordination across specialties, including HIV and obstetric care.

## Postpartum Period

PLWH face challenges in maintaining ART adherence and staying engaged in HIV care post-partum. While pregnancy care guidelines promote frequent visits to optimize treatment and reduce perinatal transmission, retention rates in care after giving birth are low. A cohort study in Philadelphia showed that only 39% of PLWH were retained in care during the first year postpartum, dropping to 25% by year two [64]. Retention in HIV care in the postpartum period is particularly low for people in the South and those with PHIV, with rates ranging from 23–46% [59, 65]. Early postpartum care is crucial for long-term health, as regular visits support ART adherence, management of co-morbidities, and provide an opportunity to address postpartum contraception counseling and reproductive planning. Postpartum retention in care is particularly crucial for PLWH who desire to breast/chestfeed, as maternal viral load monitoring is required to reduce the risk of HIV acquisition in breastfed infants.

Clinical considerations for PLWH in the postpartum period center on addressing factors impacting ART adherence and retention in HIV care, such as mental health, substance use care, HIV disclosure, social support, competing priorities with infant health and social determinants of health. The use of long-acting injectable ARVs postpartum represents an opportunity for those who struggle with oral ART, but this strategy demands bimonthly visits to clinics which may not be feasible in the postpartum period. Individualized care plans and case management, particularly for parents who desire to breast/chestfeed, need to be established and reviewed before labor and delivery to facilitate the transition to the postpartum period.

## Updated Infant Feeding Guidelines

In early 2023, U.S. Department of Health and Human Services (DHHS) Guidelines revised its recommendations around infant feeding practices for PLWH [66]. Prior to this change, formula feeding was recommended to prevent any chance of transmission through human milk [7]. The American Academy of Pediatrics and the US Centers for Disease Control and Prevention soon after the DHHS guidelines were revised formally supported this approach [67, 68]. While replacement feeding eliminates any risk of transmission, in lactating persons with consistent and persistent viral suppression on ART, the risk of infant HIV acquisition is very low. The Promoting Maternal-Infant Survival Everywhere (PROMISE) study demonstrated that among postpartum persons receiving ART during breastfeeding, the rate of breastfeeding transmission was 0.3% (95% Confidence Intervals (CI) 0.1,0.6) at six months and 0.6% at 12 months [69]. With this low risk of transmission, some mothers may feel that the potential benefits of breastfeeding may exceed risks.

Although the guideline change allows pregnant and postpartum PLWH the opportunity to make a choice about the infant feeding practices that best reflects their values and life circumstances, there remain implementation challenges, including some of those outlined in Table 3. Ideally a multi-disciplinary health care team including adult and pediatric HIV specialists, obstetric clinicians, inpatient obstetric nurses, general pediatricians, and lactation specialists jointly working together should provide pregnant and postpartum individuals with consistent information to support shared decision-making. However, not all health systems in the US provide the structure for longitudinal multidisciplinary support of pregnant and postpartum PLWH and their infants, particularly when there is need for coordination of care to support exclusive breastfeeding with appropriate support and risk-mitigation strategies. For example, as a pediatrician conducts well-child visits with infants born HIV-exposed who are breastfeeding, it is essential that they understand issues such as parental adherence to ART, parental HIV viral load results and timing, and breast health since these are of critical importance to shared decision-making around the child’s care. These multifaceted discussions can be challenging given time constraints imposed on primary care visits.

**Table 3:** Common questions related to infant feeding for individuals with HIV in the United States

Questions	Answers and related data
Why do guidelines no longer recommend against breastfeeding for people living with HIV?	-Breastfeeding provides benefits to the mother and infant that are not possible with other types of feeding [71] -Compared to infants born to parents who are HIV-seronegative, infants whose birthing parent is living with HIV are more vulnerable to infections and other complications that can be mitigated with the use of human milk [72] -People affected by HIV have strongly advocated for infant feeding choice [73–75] -The risk of HIV transmission through breastfeeding can be made very low (but not zero) through effective maternal antiretroviral treatment (ART) and/or infant prophylaxis [69]
Does an undetectable plasma viral load mean there is no virus in breastmilk?	-Lactating people with undetectable plasma HIV RNA levels frequently, but not always, also have undetectable plasma HIV RNA in their milk [76] -Subclinical mastitis alters the content of breastmilk and has been associated with an increase in breastmilk viral load [77, 78]
What is the evidence that mixed feeding (i.e., offering both breastmilk and infant formula or other foods) is dangerous in the context of parental HIV?	-When the breastfeeding parent is not on suppressive ART, infant acquisition of HIV is significantly more likely with mixed feeding than with exclusive breastfeeding [79, 80] -During the second six months of life, when complementary feeds are routinely given to breastfeeding infants, the risk of HIV transmission is not higher than during the first six months of primarily exclusive breastfeeding, when the breastfeeding parent is on suppressive ART, or the infant is on daily nevirapine -Limited data are available on mixed feeding in the first few months of life when the parent is on suppressive ART. The newborn gut may be more susceptible to viral entry with irritation from substances other than human milk
How often should the parent and infant be monitored during breastfeeding?	-There are no data that inform the frequency of viral load testing for a breastfeeding parent -Frequent monitoring during breastfeeding offers opportunities to optimize preventative strategies and offer support -There are no data that inform frequency of HIV testing for a breastfeeding infant. However, early detection of HIV in an infant offers the opportunity to start suppressive ART early which reduces viral reservoirs and improves long-term outcomes. Early detection of HIV in an infant also offers the opportunity to start pneumocystis prophylaxis, which can be lifesaving
Should infants receive prophylaxis during breastfeeding if the parent’s viral load is undetectable?	-Current DHHS guidelines do not recommend for or against infant prophylaxis throughout the period of breastfeeding [1] -Giving daily nevirapine prophylaxis to an infant during breastfeeding from a parent who is not taking ART results in the same low rates of infant HIV (~ 0.3% over 6 months) as does suppressive ART to the parent [81] -Rates of adverse events in infants receiving 6 months of daily nevirapine prophylaxis are not different from rates of adverse events in infants receiving placebo [82]
What options are available for a breastfeeding parent with a detectable HIV viral load?	-Current DHHS guidelines recommend that breastfeeding be temporarily or permanently halted if the viral load is detectable or if the breastfeeding parent develops mastitis [66] -Pumping and flash pasteurizing the breastmilk can eliminate HIV from breastmilk -Introducing or increasing infant prophylaxis can reduce risk of HIV transmission
When is the optimal time to wean?	-While guidelines do not specify the approach to weaning, longer duration of breastfeeding is associated with longer need for ongoing shared decision making/potential ongoing risk of HIV transmission −6 months exclusive breastfeeding is recognized as a best practice when a postpartum person is HIV seronegative -Beyond 6 months, breastmilk continues to offer health benefits for the parent and infant, and is particularly important if a parent does not have consistent access to replacement formula

The revised guidelines have been welcomed by pregnant and postpartum PLWH [70]. However, data are needed to further refine recommendations. For example, there are no data to guide recommended frequency of viral load testing for the parent or HIV testing of the infant. While this can be negotiated in the course of shared decision-making discussions, studying the heterogeneous approaches that are likely to result should help future guidelines to recommend approaches that maximize safety and minimize family burden. It would be beneficial to maintain a registry of infant feeding choice that comprehensively characterizes contributions to care for families choosing breastfeeding including frequency and results of monitoring, parental and infant ARVs, approaches to counseling, choices made in the face of complications such as mastitis, and outcomes. Such a registry could identify opportunities to further refine guidelines and may provide an opportunity to measure and improve health equity.

## Conclusion

The use of ART during pregnancy has transformed the landscape of HIV management in pregnancy, nearly eliminating perinatal HIV transmission in settings with robust healthcare systems. Continued research, global cooperation, and tailored interventions are essential to address remaining challenges. By prioritizing maternal and infant health, ART not only prevents transmission but also fosters a brighter future for families affected by HIV.

## Key References

- Panel on Treatment of HIV During Pregnancy and Prevention of Perinatal Transmission. Recommendations for the use of antiretroviral drugs during pregnancy and interventions to reduce perinatal HIV transmission in the United States. Department of Health and Human Services. Available at: https://clinicalinfo.hiv.gov/en/guidelines/perinatal. Accessed (Awaiting new guidelines)
  - ⚬ This guideline is published annually and provides the most up to date management of pregnant persons living with HIV
- Antiretroviral Pregnancy Registry Steering Committee. Morrisville, NC: Registry Coordinating Center, 2024. Available at: www.APRegistry.com. Accessed January 3, 2025.
  - ⚬ This report is published semi-annually and summarizes the current state of evidence for teratogenic effects for pregnancies exposed to antiretroviral medication
- Abuogi L, Noble L, Smith C; COMMITTEE ON PEDIATRIC AND ADOLESCENT HIV; SECTION ON BREASTFEEDING. Infant Feeding for Persons Living With and at Risk for HIV in the United States: Clinical Report. Pediatrics. 2024 Jun 1;153(6):e2024066843. https://doi.org/10.1542/peds.2024–066843. PMID: 38,766,700.
  - ⚬ a recent publication from the American Academy of Pediatrics discussing Infant feeding for Persons Living with and at Risk for HIV in the United States

## Data Availability

No datasets were generated or analysed during the current study.
